# Multi-Modality Microscopy Image Style Augmentation for Nuclei Segmentation

**DOI:** 10.3390/jimaging8030071

**Published:** 2022-03-11

**Authors:** Ye Liu, Sophia J. Wagner, Tingying Peng

**Affiliations:** 1 Department of Mathematics, Technical University Munich, 85748 Garching, Germany; yeliu0724@hotmail.com (Y.L.); sophia.wagner@helmholtz-muenchen.de (S.J.W.); 2 Helmholtz AI, Helmholtz Munich, 85764 Neuherberg, Germany

**Keywords:** style transfer, data augmentation, nuclei segmentation

## Abstract

Annotating microscopy images for nuclei segmentation by medical experts is laborious and time-consuming. To leverage the few existing annotations, also across multiple modalities, we propose a novel microscopy-style augmentation technique based on a generative adversarial network (GAN). Unlike other style transfer methods, it can not only deal with different cell assay types and lighting conditions, but also with different imaging modalities, such as bright-field and fluorescence microscopy. Using disentangled representations for content and style, we can preserve the structure of the original image while altering its style during augmentation. We evaluate our data augmentation on the 2018 Data Science Bowl dataset consisting of various cell assays, lighting conditions, and imaging modalities. With our style augmentation, the segmentation accuracy of the two top-ranked Mask R-CNN-based nuclei segmentation algorithms in the competition increases significantly. Thus, our augmentation technique renders the downstream task more robust to the test data heterogeneity and helps counteract class imbalance without resampling of minority classes.

## 1. Introduction

The evaluation of cell-level features is a key task in the histopathological workflow. Features, such as nuclei shape and distribution, are used to determine cell, tissue, and cancer types and are therefore relevant for the clinical diagnosis of cancer, e.g., for cancer identification, grading, and prognosis [1]. Deep learning serves as a promising tool to quantify these features in an automated manner [2]. However, the performance of these models depends heavily on the quality and quantity of training data since they require accurate and time-consuming segmentation masks. To tackle this, the Kaggle competition 2018 Data Science Bowl (DSB) (https://www.kaggle.com/c/data-science-bowl-2018 (accessed on 15 January 2022)) was initiated to find segmentation algorithms that are robust and effective in various microscopy set-ups, cell lines, and, most significantly, types of light microscopy. The nuclei presented in the images are from a variety of creatures, including humans, mice, and flies. In this dataset, most of the images are stained primarily with DAPI or Hoechst. Hematoxylin and eosin-stained tissue samples are also included in the dataset. Among 3891 submissions during the challenge, the top-performing methods were mostly based on the common segmentation network architectures, Mask R-CNN [3] and U-Net [4]. The most important factors influencing the competition ranking were the amount of training data (some teams also used private training data aside from the data provided by the challenge organizer), complex pre-processing and post-processing, and ensemble learning. A common problem that encumbers the performance is the dataset’s bias: while segmenting fluorescent images, which make up most of the training set, is relatively easy and accurate, segmenting less-represented image types, such as bright-field images of stained tissue sections, is difficult and inaccurate [5].

Style transfer has been widely used for image synthesis from one modality to another, e.g., from computed tomography (CT) to magnetic resonance imaging (MRI) [6], from MRI to CT [7], from MRI to Positron Emission Tomography (PET) [8]. It has also been shown to be effective for histological images, e.g., for stain color augmentation [9]. In terms of style transfer architectures, conditional GAN (cGAN) [10] can be used for aligned image pairs and its generator can generate images of a certain class. For instance, Pix2pix [11] is a type of cGAN for paired image translation, in which the input image is conditional on the output pair. It receives a whole image as input rather than noise and class vector. The discriminator of Pix2Pix [11], called PatchGAN, generates a matrix of classifications instead of a single output for optimization. Additionally, Pix2pix [11] changed the generator to a U-Net [4] architecture. CycleGAN [12], on the other hand, introduces the cycle-consistency loss to overcome the dependency of paired images. It can be trained on two independent datasets. Unlike CycleGAN which uses a single encoder, DRIT++ [13] introduces a structure with two encoders and is capable of decomposing the image into domain-invariant content and domain-specific feature encoding. Another well-known architecture is StyleGAN [14], which increases the diversity of outputs and has more control over image features by better disentangling the latent factors of variations.

Among the top-25 submissions in the competition, the team BIOMAGic introduced the only approach using style transfer. They refined their approach after the competition and named it NucleAlzer [15]. In addition to the DSB dataset, they included annotated data from twelve data sources increasing the number of training images from 735 to 1102 and the number of annotated nuclei from 33,814 to 80,692. For style transfer, they trained a Pix2pix [11] style transfer network (from the original image to segmentation mask) for each cluster of similar images in the unannotated data, resulting in a total of 134 networks. With these, 20 synthetic image/mask pairs of each style with nuclei at certain places are generated and added to the training set to train Mask R-CNN [3] for instance segmentation. The winner [5] of the competition used an ensemble technique of a total of 32 models based on eight different model architectures. Pretrained Resnets, Dual Path networks, and Inception-Resnets are used as encoders and U-Net or feature pyramid networks (FPN) [16] as decoder architectures. The second-best approach used a single pretrained FPN-based neural network model and two customized output layers. A multitask framework is used to train the customized output layers. Additionally, they introduced a new loss function that penalizes the instance errors based on the object’s size.

In this paper, we propose to facilitate network training by a GAN-based style transfer data augmentation technique. By synthesizing less-represented image types from well-represented ones, our style augmentation increases the number of images of minority types in the training set. More specifically, we use disentangled representations in the style transfer, i.e., we decompose the input into its content and style representations and only alter the style without changing the content. Since the structure is thereby preserved, the style augmented images have the same nuclei locations and shapes as the original image. Therefore, our augmentation technique presents the segmentation network a multitude of styles already during training, rendering it more robust to the heterogeneity in the test data. Our experiments on the DSB challenge dataset demonstrate that by increasing the variety in the training dataset, our augmentation technique improves the overall segmentation accuracy.

## 2. Materials and Methods

**Figure 1:**
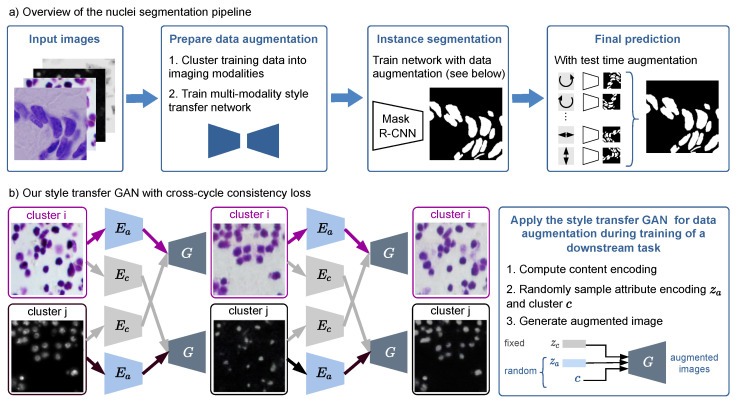
(**a**) Overview of our nuclei segmentation pipeline with multi-modality style transfer as data augmentation, Mask R-CNN as instance segmentation network, and test time augmentation for the final prediction. (**b**) Our style transfer GAN is trained with a cross-cycle consistency loss that does not need paired data. The right box shows how it is applied for data augmentation during training of the nuclei segmentation network.

Our nuclei segmentation workflow consists of three steps: data augmentation (split into clustering and training of a style transfer network), training of an instance segmentation network, and evaluating the segmentation network with test time augmentation (see Figure 1). In the following, we detail each of the three steps.

### 2.1. Dataset

**Figure 2:**
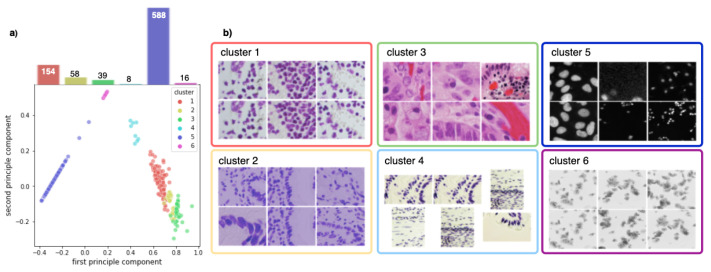
(**a**) PCA decomposition of hue saturation values of all training images and their class distribution. Each point represents one image, colored according to the corresponding cluster. (**b**) Exemplary images for each cluster.

The dataset was provided by the competition organizers and is publicly available in the Broad Bioimage Benchmark Collection with access number BBBC038 (https://bbbc.broadinstitute.org/BBBC038 (accessed on 15 January 2022)). It is divided into three sets: a training set of 670 images with nuclei segmentation masks provided (29,464 nuclei), a first-stage test set of 65 images (4152 nuclei), and a second-stage test set, which includes 3019 images (37,333 nuclei). The evaluation of nuclei segmentation for the annotated first-stage test set was disclosed later by the organizers. However, the official evaluation and ranking of a segmentation algorithm are based on the performance of the second-stage test set, which hides the annotated nuclei masks from the participants. Both training and test images are highly varied in terms of large experimental settings such as lighting conditions, cell lines, nuclei densities, and microscopy imaging modalities (with a few examples shown in Figure 1a and Figure 2). It is worth noting that the test set is not only substantially larger than the training set, but also includes novel image modalities rendering the segmentation task extremely challenging.

### 2.2. Data Augmentation Step I: Clustering Training Images into Different Modalities

Since the dataset does not contain any metadata that can be used to determine the image modalities, we first divide our training images into multiple domains to perform image style transfer. Each cluster should ideally correspond to one imaging modality. We used the classic K-means algorithm to divide the training images into six distinct clusters based on their hue saturation value (HSV). Furthermore, we observed that the fluorescent images are typically dark and have low contrast. So we use the Contrast Limited Adaptive Histogram Equalization (CLAHE) (https://scikit-image.org/docs/dev/auto_examples/color_exposure/plot_equalize.html (accessed on 15 January 2022)) for contrast enhancement. For each section of an image, CLAHE computes a contrast histogram and adjusts the local contrast accordingly if that section is darker or brighter than the rest of the image.

### 2.3. Data Augmentation Step II: Multi-Modality Style Transfer

As shown in Figure 1b, for two exemplary images from different domains, the modality transfer model based on [13] first decomposes each image into a domain-invariant content encoding using the content encoder $E_{c}$ and a domain-specific attribute encoding using the attribute encoder $E_{a}$, respectively. The content encoding and the attribute encoding, together with the domain information, can then be used to generate a synthetic image, where the structure is preserved. To create various synthetic images as augmentation during training of the segmentation network, we sample attribute encoding and domain vector randomly, while leaving the content-encoding fixed (see Figure 1b). We apply this augmentation technique randomly to half of the training images additionally to standard augmentations.

The underlying assumption of the style-transfer model is that the content space is shared across the domains. Therefore, during training, the content vector $z_{c}$ can be exchanged between the modalities. Since the dataset is unpaired, i.e., we do not have ground truth for the style transfer, we use a cross-cycle consistency loss $L_{cc}$ for training (see Figure 1b): The content encoding of two images from different modalities is swapped and a first image translation is generated. Subsequently, the translation is repeated, again by computing the encodings and then swapping the content encoding. The second image translation should resemble the original input, which is enforced by applying a loss based on the $L_{1}$-norm. A classical adversarial loss $L_{d}$ to enforce realistic synthetic images and an adversarial loss on the content encoder $L_{c}$ to eliminate domain-specific information in the content encodings are also applied. Additionally, an $L_{1}$-loss for image reconstruction, $L_{\mathrm{recon}}$, is applied: the original image is decomposed into its content and attribute encodings and then reconstructed using those representations. To enable sampling from the latent space, $L_{\mathrm{latent}}$ is an $L_{1}$-loss for latent space reconstruction, and the loss $L_{KL}$ enforces the latent attribute space to be distributed according to a prior Gaussian distribution. Please refer to [13] for a detailed explanation of each loss. The final objective function is given by

(1) $$\begin{array}{ccc} L_{\mathrm{total}} & = & w_{cc}L_{cc}+w_{c}L_{c}+w_{d}L_{d}+w_{\mathrm{recon}}L_{\mathrm{recon}}+w_{\mathrm{latent}}L_{\mathrm{latent}}+w_{KL}L_{KL}, \end{array}$$

where the parameters *w* are used to weight the loss terms.

Since the content encoding is fixed during augmentation, the augmented image has the same nuclei locations and shapes as the input image and thus inherits the nuclei segmentation mask from the original image. This is a key difference between our approach and a common CycleGAN-based image style transfer [12], as there is no guarantee of content invariance after the CycleGAN style transfer.

### 2.4. Mask R-CNN for Instance Segmentation

For nuclei segmentation, we used two implementations published in the competition leaderboard (https://github.com/Lopezurrutia/DSB_2018 (accessed on 15 January 2022)) (https://github.com/mirzaevinom/data_science_bowl_2018 (accessed on 15 January 2022)) as baselines. Both teams use Mask R-CNN [3] for segmentation. The implementations differ in the image pre-processing and preparation of the network training. We choose these two methods because of their publicly accessible code, sufficient documentation, excellent performance (both methods were ranked among the top-5 in the leaderboard), as well as relatively simple network training (only one model needs to be trained, by contrast, the top-1 approach trained 32 models, which would consume considerably more training time and resources). Notably, the second approach developed by the team mirzaevinom. used additional data beyond the data provided by the organizer. However, we only used the annotated training data provided by the organizer to train our model, so our model performance differs slightly from their results reported in the leaderboard. For each method, we train two models, one with and one without multi-modality style transfer as augmentation technique.

### 2.5. Test Time Augmentation

In the inference stage, we use test time augmentation (TTA). TTA applies simple augmentations on the test images, e.g., rotating, flipping, color jittering, and image rescaling, and aggregates the model predictions on the augmented images. Thereby, TTA improves the robustness of Mask R-CNN without any additional training cost, leading to an increase in segmentation accuracy when compared to predicting only on the original image [17].

### 2.6. Implementation Details

All models were trained on Intel Xeon 6230 system with 128 GB of RAM and a Tesla V100 graphics card. The style transfer model is trained with batch size two and a learning rate of 0.0001 for 600 epochs. For the optimizer, we used the Adam optimizer with $\beta_{1}$ 0.5 and $\beta_{2}$ 0.999 and a weight decay of 0.0001. We trained the model on five domains and applied the style transfer augmentation with probability 0.5 in the segmentation downstream task.

## 3. Results

### 3.1. Clustering into Modalities

Figure 2b shows that the training set can be clustered into six sets that correspond indeed to the different imaging modalities present in the dataset. These clusters can also be seen in the low-dimensional principal component analysis (PCA) embedding of the training data visualized in Figure 2a. We observe that the training set is highly imbalanced: over 75% of all training images are fluorescent images, whilst the remaining bright-field images are grouped into five distinct, partly very small clusters.

### 3.2. Improved Segmentation Performance by Style Transfer Augmentation

**Figure 3:**
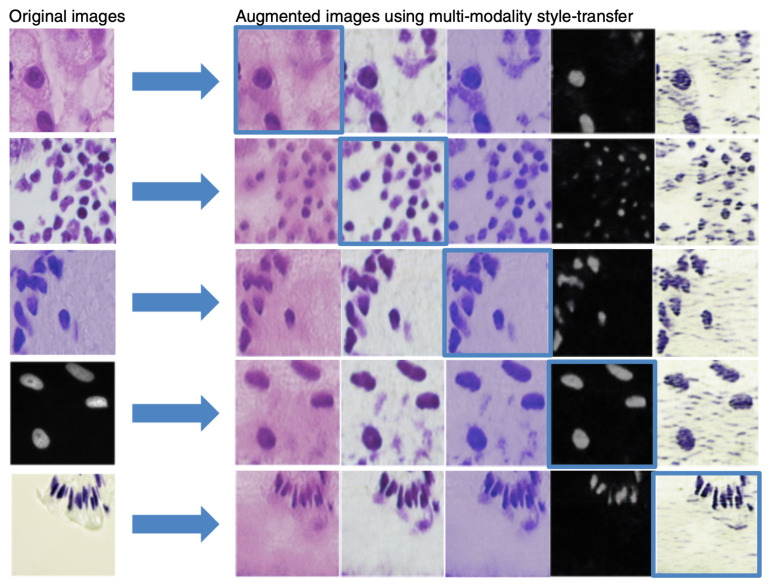
Examples of the outcome of multi-modality style transfer. **First column**: original images. **Remaining columns**: result of each domain’s multi-modality style transfer with self-reconstruction on the diagonal, highlighted in blue boxes.

Figure 3 shows a few examples of images created by our multi-modality style transfer GAN from one domain to the others. Visual assessment reveals that our generated images resemble real ones, implying that they are well suited for the augmentation task.

We quantify the add-on value of our proposed augmentation method by training models with and without augmentation of the multi-modality style transfer GAN. The final evaluation score is based on the object-level errors. If the predicted mask and segmented object have an Intersection-over-Union (IoU) score greater than a predefined threshold, they are regarded as correctly identified. The official competition metric *S* computes a precision score for semantic segmentation by evaluating true positives (TP), false positives (FP), and false negatives (FN) at several IoU thresholds *t* and then averaging over these, i.e.,

(2) $$\begin{array}{c} S=\frac{1}{\left|T\right|}\sum_{t\in T}\frac{\mathrm{TP}(t)}{\mathrm{TP}(t)+\mathrm{FP}(t)+\mathrm{FN}(t)},\;\mathrm{where}\;T=\left\{0.1,0.15,\cdots ,0.95\right\}. \end{array}$$

**Figure 4:**
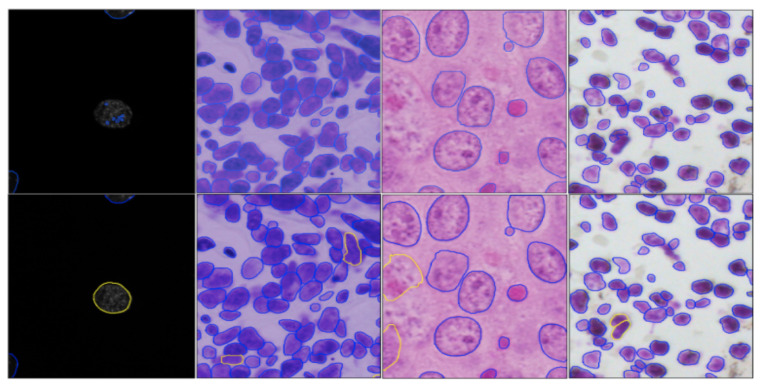
Nuclei segmentation results without (**first row**) and with (**second row**) our augmentation. Agreeing mask contours are shown in blue. Yellow contours in the second row show the improvements compared to the first row.

After that, the average of all the score is calculated over all images. By submitting our segmentation results of the second-stage test dataset to the Kaggle competition (https://www.kaggle.com/c/data-science-bowl-2018/submit (accessed on 15 January 2022)), we obtain the final evaluation score. As shown in Table 1, including style transfer augmentation in the method increased the nuclei segmentation accuracy from 53.2% to 60.9% for the method Deep Retina and 59.9% to 61.3% for method Inom Mirzaev, respectively. Notably, a score around 61% was ranked among the top-5 and almost only achieved by using additional datasets. In Figure 4, we can observe that our augmentation technique using the multi-modality style transfer GAN improves the segmentation results, especially in less-represented image modalities such as bright-field images of stained tissue sections and fluorescent images of large nuclei (most fluorescent images in the training set contain only small nuclei).

In addition to the above two baseline methods, we also quote the result from the team BIOMAGic (57.0% IoU), as it was the only one to use style transfer among the top-25 submissions during the competition. Unlike our style transfer between multiple microscopy image modalities, BIOMAGic (and its successor nucleAIzer [15]) performed style transfer between images and their segmentation masks, which can be trained only by annotated images. In contrast, our method can be trained in an unsupervised fashion using images without annotated segmentation masks. In particular, their style transfer approach required training 134 networks for fine-grained image clusters for the same competition, whereas our approach only required the training of one network.

## 4. Discussion

**Figure 5:**
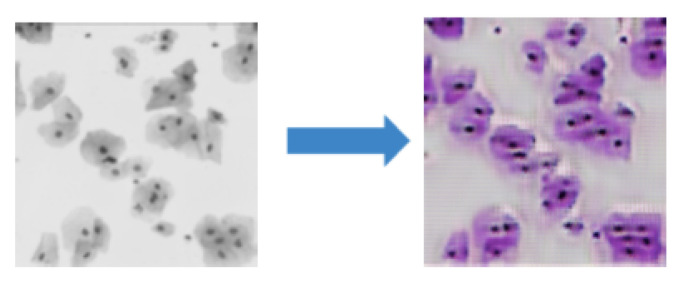
The style transfer GAN fails when images at two modalities contain different content.

Although our approach is effective in transferring style across the majority of modalities, it also has limitations. When using our style transfer GAN from image modalities that only show nuclei to another modality where both nuclei and cytoplasm are visible, unrealistic images are generated as demonstrated in Figure 5. This is because the underlying assumption of our model is violated. In particular, these two modalities do not only differ in style, but also in content; hence, the content and style cannot be efficiently disentangled.

Our style transfer model is built based on the DSB dataset, which mostly consists of images from fluorescence microscopy, primarily stained with DAPI or Hoechst, but also includes tissue samples stained with hematoxylin and eosin. Currently, our model is able to transfer the style between the five modalities contained in this dataset. Generally, it can be trained on all kinds of imaging modalities that share the same morphological structure and thereby fulfill our training assumption. Apart from the standard microscopy approaches above, our work can also be applied to images acquired by alternative microscopy technologies such as lens-free microscopy, a more cost-effective option that does not require expensive optical lenses and has a wider field of view. As a result, a much more affordable microscopy technique capable of monitoring cells continuously within an incubator and ideal for low-cost point-of-care devices aiming at resource-limited settings [18]. Our future work will involve transferring the style of lens-free microscopy images to the existing modalities.

## 5. Conclusions

In summary, we developed an augmentation technique using a multi-modality style transfer GAN to transfer microscopy nuclei images from one modality to another. During training a Mask R-CNN for nuclei segmentation, this augmentation strategy facilitates the training by increasing the diversity of the training images, hence making it more robust to the test data heterogeneity and resulting in better segmentation accuracy.

## Figures and Tables

**Table 1:** Evaluation scores of different segmentation methods.

Method	Score [↑]
BIOMAGic	0.570
Deep Retina w/o our augmentation	0.532
Deep Retina w/ our augmentation	0.609
Inom Mirzaev w/o our augmentation	0.599
Inom Mirzaev w/our augmentation	0.613

## Data Availability

The data that support the findings of this study are available in the public domain: Broad Bioimage Benchmark Collection at https://bbbc.broadinstitute.org/BBBC038/ (accessed on 15 January 2022) with accession number BBBC038.
